# Enzymes Dysregulation in Cancer: From Diagnosis to Therapeutical Approaches

**DOI:** 10.3390/ijms241813815

**Published:** 2023-09-07

**Authors:** Valentina Pozzi, Roberto Campagna, Davide Sartini, Monica Emanuelli

**Affiliations:** 1 Department of Clinical Sciences, Polytechnic University of Marche, 60131 Ancona, Italy; d.sartini@univpm.it (D.S.); m.emanuelli@univpm.it (M.E.); 2 New York-Marche Structural Biology Center (NY-MaSBiC), Polytechnic University of Marche, 60131 Ancona, Italy

The metabolic reprogramming that occurs in cancer cells is a hallmark of cancer [1]. Indeed, elucidating the effects of dysregulated metabolism on cancer cell phenotype constitutes the focus of many researchers. Proliferating malignant cells are highly demanding in terms of energy supply and molecules required to fuel biosynthesis. Thus, cancer cells display significant metabolic alterations to meet the requirements for rapid growth and survival. For instance, cancer cells are characterized by enhanced glycolysis, the so-called “Warburg effect”, and glutamine catabolism. The reprogramming of cell metabolism toward a malignant phenotype is the result of a series of downstream events triggered by the mutations of oncogenes or tumor suppressors, dysregulated signal transduction pathways, and altered nutrient availability in the tumor microenvironment [2,3].

Despite the continuous accumulation of mutations responsible for intra- and inter-tumor heterogeneity, the dysregulation of specific enzymes can be a distinguished feature for some groups of malignancies [4,5,6]. Among the enzymes that play a key role in the proliferation and aggressiveness of malignant cells there are glycolytic enzymes like lactate dehydrogenase (LDH), caspases, cyclin-dependent kinases, redox–detox enzymes and NAD^+^-dependent enzymes [7,8]. In recent years, several enzymes emerged as potential biomarkers for the diagnosis or prognosis of several malignancies [9,10,11,12,13,14]. Moreover, the possibility of designing specific inhibitors has also opened the possibility of utilizing these biomarkers as therapeutic targets [15,16,17,18]. The need to discover novel enzymatic pathways dysregulated in cancer, as well as identifying novel potential therapeutic targets for cancer management, was highlighted by several papers published in this Special Issue.

The works published by Tossetta et al. reviewed the pathophysiology, treatment options and novel biomarkers in ovarian cancer. In particular, authors focused on the dysregulated enzymatic pathways responsible for the enhanced chemoresistance of ovarian cancer cells. The authors focused on the role of metformin, which exerts important anti-cancer effects in malignant cells as an inhibitor of respiratory chain complex I, which in turn oxidizes the NADH yielded through the Krebs cycle. Moreover, it has been demonstrated that this molecule significantly restores drug sensitivity in ovarian cancer cells that display resistance to paclitaxel and platinum-derived drugs [19].

The same research group reviewed the effect of the antioxidant enzyme NAD(P)H:quinone oxidoreductase 1 (NQO1), whose expression is mainly regulated by nuclear factor erythroid 2-related factor 2 (NRF2), a transcription factor that plays a key role in ovarian cancer onset and progression [20,21]. Authors underlined how the NQO1 enzyme could be a promising therapeutic target in ovarian cancer since it plays a pivotal role in ovarian cancer progression and chemotherapeutic response, utilizing glutaminase inhibitors such as CB-839 to suppress NRF2/NQO1-mediated pathways and enhance the chemosensitivity of ovarian cancer cells [22].

Chen et al. reviewed the current literature about the impact of glutathione peroxidase 4 (GPX4) on cancer cell ferroptosis, a form of iron-dependent cell death consequent to high iron accumulation and lipid peroxidation. The authors reported how GPX4 displays an excellent antioxidant capacity in preventing ferroptosis. This is in line with the evidence that GPX4 expression levels are higher in several types of cancer cells compared to normal tissues, allowing cells to counteract ferroptosis more efficiently. For these reasons, the inhibition of the GPX4 pathway could represent an effective strategy for anti-cancer therapy. Thus, there is ongoing research that aims to characterize efficient GPX4 inhibitors for clinical tests [23].

In an interesting experimental work, Dalpatraj et al. investigated the use of GSK-J4, a H3K27 demethylase (JMJD3/KDM6B) inhibitor to study its effects on TGFβ-induced epithelial-to-mesenchymal transition (EMT) in prostate cancer cells. Tests were either performed alone or in combination with phytochemical hesperetin, a citrus bioflavonoid. The researchers found that coupling hesperetin and GSK-J4 treatment at lower doses effectively prevented TGFβ-induced migration and the invasion of the prostate cancer cells, effects that were associated with epigenetic modifications [24].

Manna et al. demonstrated with an elegant study that the steroidogenic acute regulatory (StAR) protein is differentially expressed in tumor and non-tumor human and mouse breast cells/tissues, highlighting the potential role of this cholesterol transporter as a novel diagnostic biomarker for breast cancer. Moreover, they demonstrated that a number of histone deacetylase inhibitors (HDACIs) were efficient in suppressing StAR and also estrogen levels in vitro. This was found not only in hormone-sensitive human breast cancer cells, but also in primary cultures of breast cancer epithelial cells, suggesting StAR’s possible therapeutic utility as a drug target for the treatment of breast cancer [25].

Finally, Lucia et al. elaborated a complex but novel thermodynamic approach to the epigenomics of cancer metabolism based on the consumption of metabolites to reverse the membrane electric potential required to sustain cell activity, a process driven by ion fluxes. Furthermore, they analytically demonstrated the correlation between cell proliferation and membrane electric potential through a thermodynamic approach, emphasizing how its modulation is associated with the inflow and outflow of ions [26].

Altogether, this Special Issue collected interesting experimental and review articles that will be of interest to the scientific community.
